# Who wants to join preventive trials? – Experience from the Estonian Postmenopausal Hormone Therapy Trial [ISRCTN35338757]

**DOI:** 10.1186/1471-2288-5-12

**Published:** 2005-04-12

**Authors:** Sirpa-Liisa Hovi, Matti Hakama, Piret Veerus, Mati Rahu, Elina Hemminki

**Affiliations:** 1 National Research and Development Centre for Welfare and Health, STAKES, Health and Social Services, PO Box 220, FI-00531 Helsinki, Finland; 2 School of Public Health, University of Tampere, FI-33014 Tampere, Finland; 3 Department of Epidemiology and Biostatistcs, National Institute for Health Development, Hiiu 42, 11619 Tallinn, Estonia; 4 Estonian Center of Excellence in Behavioral and Health Sciences, Tartu-Tallinn, Estonia

## Abstract

**Background:**

The interest of patients in participating in randomized clinical trials involving treatments has been widely studied, but there has been much less research on interest in preventive trials. The objective of this study was to find out how many women would be interested in a trial involving postmenopausal hormone therapy (PHT) and how the women's background characteristics and opinions correlated to their interest.

**Methods:**

The data come from recruitment questionnaires (n = 2000) sent to women in Estonia in 1998. A random sample of women aged 45 to 64 was drawn from the Population Registry. The trial is a two-group randomized trial comparing estrogen-progestogen therapy with placebo or no drugs. A brief description of the study was attached to the questionnaires. Women were not told at this stage of the recruitment which group they would be assigned to, however, they were told of the chance to receive either hormone, placebo or no treatment.

**Results:**

After two reminders, 1312 women (66%) responded. Eleven percent of the women approached (17% of the respondents) were interested in joining the trial, and 8% wanted more information before deciding. When the 225 women who stated clearly that they were interested in joining and the 553 women who said they were not interested were compared, it was found that interested women were younger and, adjusting for age, that more had given birth; in other respects, the sociodemographic characteristics and health habits of the interested women were similar to those of the non-interested women. The interested women had made more use of more health services, calcium preparations and PHT, they were more often overweight, and more had chronic diseases and reported symptoms. Interested women's opinions on the menopause were more negative, and they favoured PHT more than the non-interested women.

**Conclusion:**

Unlike the situation described in previous reports on preventive trials, in this case Estonian women interested in participating in a PHT trial were not healthier than other women. This suggests that trials involving PHT are more similar to treatment trials than to preventive trials. In a randomized controlled trial, more information should be obtained from those women who decline to participate.

## Background

Randomized controlled trials (RCTs) are needed so as to avoid bias in scientific research. There are many studies and reviews on factors that promote or hinder interest in participating in treatment trials – both in regard to patients and physicians [see e.g. [1-4]]. Men, patients who are older, less educated and from lower socioeconomic backgrounds, non-whites, smokers, and persons lacking adequate social support are more willing to participate in clinical trials. Furthermore, they tend to be more severely ill than those who do not participate. Identified obstacles include patients' and physicians' disapproval of patients' serving as research subjects, a lack of altruistic motives, distrust of the medical profession, a lack of knowledge of what is required of trial participants, and preference for a certain treatment. Many patients do not understand the reasons why treatments should be allocated at random, and this is an important reason why patients choose not to join randomized clinical trials. [5-8]. Disinterest on the part of patients and physicians in participating in clinical trials constitutes a threat to the generalizability of RCTs [9].

There has been much less research on participation in trials studying preventive measures (preventive trials). The data available thus far suggest that there is a difference in the types of people who join preventive trials and treatment trials. Participants in preventive trials tend to be better off than non-participants as regards socioeconomic situation, health habits and health [10-14]. However, most evaluative studies have failed to document adequately the characteristics of persons who were eligible but did not participate [1]. Less is known of physicians' motivation as regards including or encouraging people to participate in preventive trials [15]. Preventive drug therapy, and thus trials involving such drugs, are likely to increase in the future, and more information is needed on who wants to take part in preventive trials. Such data are useful in order to increase the recruitment rate as well as to interpret trial results.

By means of a mailed questionnaire in the Estonian Postmenopausal Hormone Therapy (EPHT)-trial, we recruited healthy 45–64-year-old Estonian women for studying the long-term (5-year) health effects of PHT. The trial investigated the immediate effects of PHT on well-being and symptoms, impacts on the experience of the climacteric, on aging and partner relationships, and influences on the use of health services. Furthermore, the trial investigated the placebo effect and trial effect by means of the design as well as their effect on recruitment and compliance. The object of this paper is to report on how many women were interested in participating in such a trial and how sociodemographic characteristics, health, health habits, health services utilization and opinions regarding the menopause and aging influence women's interest.

## Methods

The trial is a two-group randomized trial comparing oestrogen-progestogen therapy to placebo or no drugs carried out in Estonia. The participants were allocated to four random arms forming two groups: the blind group was given an active drug or a placebo, and the non-blind group was given an active drug or no treatment. Women were informed that drugs would be provided for five years. A random sample of women aged 45 to 64 was drawn from the Population Registry and questionnaires were sent to the women. A brief description of the study was attached to the questionnaire and women were invited to participate in the trial if they were found to be eligible. In the letter, women were told about the sampling; that the trial investigated the health problems of perimenopausal and older women in Estonia, especially the long-term effect of hormonal replacement therapy after cessation of periods; and that the drugs would be provided for five years. In this first letter, women were not presented with the trial design in detail, but they were told that the women will be randomly divided into groups of hormone treatment, placebo, or no tablets. Further, they were told that physician examinations will be provided annually, and the possible risks and benefits of PHT were explained. The study plan had been accepted in the Committee of Medical Ethics in Tallinn. The first 2000 women, in the sample of spring 1998, were sent a more detailed questionnaire, which provided more information on the respondents. After two reminders, the response rate was 66% (n = 1 312).

The questions used in this article were structured, with fixed alternatives. As regards current health, women were asked to choose between very good, good, average, poor or very poor; "very good" and "good" were later combined to "good". To measure health status, women were asked if they had or had had chronic diseases, such as cancer (breast, uterus, ovary), myocardial infarction, cardiac failure, hypertension, stroke, thromboses, liver diseases, renal failure, diabetes or icterus. In the case of other symptoms, women were given a checklist of 18 different symptoms or health problems and asked to choose all that they had experienced within the past two weeks. In the case of health habits, questions on current smoking, alcohol consumption and exercise were asked. The choices for current smoking were: no, yes every now and then, and yes daily, how many cigarettes per day. The choices for alcohol consumption were not at all, low, moderate, fairly high and high. Exercise in leisure-time was elicited using the choices not at all, a little, some, a lot, a large amount; "a lot" and "a large amount" were later combined to "plenty of exercise". Body mass index (BMI) was calculated by dividing weight in kilograms by the square of height in meters.

Women's opinions on the menopause were elicited using the statements: "The menopause is a normal phase in a woman's life, and usually it does not need treatment by a doctor", and "A woman does not loose her femininity during the menopause". Women's opinions concerning PHT were elicited using the statements: "PHT effectively prevents osteoporosis ", "PHT should be given to all middle-aged women with (menopausal) symptoms", and "PHT should be given to most postmenopausal women". The possible answers were: I totally agree, I agree somewhat, I don't know, I disagree somewhat, and I totally disagree. In the analyses, the choices "I totally agree" and "I agree somewhat " were later combined to "agree".

After the previous statements came the remark 'the next questions deal with the menopause and PHT use. Those with normal periods and who do not use PHT may stop here'. This mistake resulted in our many missing answers concerning PHT use (see Table 2), and the percentages in Table 2 are given in two ways.

**Table 2:** Comparison of health service utilization of women interested and not interested in participating in a randomized PHT preventive trial in Estonia, proportion (%) of women, and age-adjusted odds-ratios (OR).^1)^

	Interested	Non-interested	OR (95% CI)^1)^
	(n = 225)	(n = 553)	
	%	%	
Physician visit in past year	76	68	1.53 (1.06–2.20)
Gynaecologist visit in past year	53	39	1.40 (1.01–1.94)
Used calcium drugs in last 2 weeks	32	23	1.49 (1.04–2.13)
Used PHT at some time ^2)^	26	11	2.62 (1.58–4.35)
Used PHT at some time ^3)^	16	8	2.05 (1.26–3.35)

1) Reference group: non-interested

2) Excluding missing values, interested n = 90 (40%), non-interested n = 143 (26%), see Methods

3) Including missing values in the denominator

Open-ended questions were also used to ask women what kind of positive and negative features they associated with the menopause. The proportions giving positive or negative aspects are also reported in this article.

Testing the statistical significance of medians was done using Mann-Whitney's U test. Age-adjusted odds ratios (OR) with 95% confidence intervals (CI) were calculated by logistic regression, using interested women as the reference group. SAS 8.0 was used in the analyses.

## Results

Altogether 2000 questionnaires were sent and after two reminders, 1312 women (66%) responded (Table 1). Non-respondent women were somewhat older and were somewhat more often residents of the capital than were respondents.

**Table 1:** Distribution of women by their interest in participating in a randomized PHT preventive trial in Estonia and comparisons of the background characteristics of women according to their interest in participating. ^1)^

	Interested	Want more information	Do not know	Non-interested	All respondents	No reply
	(n = 225)	(n = 163)	(n = 371)	(n = 553)	(n = 1312)	(n = 688)
	(% = 11)	(% = 8)	(% = 19)	(% = 28)	(% = 66)	(% = 34)
Median age, years ^2)^	51	53	53*	56***	54	53*
Lives in the capital, %	68	64	67	62	65	76 ^3)^
Married or cohabiting, %	62	64	63	58	61	
≥ 12 years of education, %	64	62	60	61	61	
In employment, %	78	72	73	67	70	
Given birth, %	92	92	89	85^4)^	87	

^1) ^Adjusted for age

^2) ^Statistically significant: * p < 0.05, *** p < 0.0001 compared to the "interested" group

^3) ^Age adjusted OR 0.64 (CI 0.46 0.90), reference group "interested"

^4) ^Age adjusted OR 2.00 (CI 1.16 3.46), reference group "interested"

Of the 1312 respondents, 17% wanted to participate in the trial. Using the whole sample of 2000 women as a basis for calculation, 11% were interested in participating in the trial, 42% did not want to participate,12% wanted to receive more information before deciding, and 28% gave no answer ('do not know'). (Table 1).

The socioeconomic background characteristics of these four groups were very similar, but women in the "do not know" and "non-interested" groups were older than interested women. After adjustment for age, it was found that fewer non-interested than interested women had given birth. When the women who had given a clearly positive or negative answer to the question concerning their interest in participating were compared, it was found that interested women had had more contacts with the health-care system – as measured by visits to a physician and a gynaecologist during the previous year – than had non-interested women. (Table 2). Interested women had also used calcium drugs more often, and they more often reported using PHT at some time than did non-interested women.

Health habits, smoking, alcohol use, and exercise did not vary by interest in participating (Table 3). In both groups, more than half were mildly overweight, but heavy overweight was somewhat more prevalent in those interested. Interested women more often had some chronic disease, such as hypertension, cardiac failure or diabetes. (Table 3). There was no difference in regard to subjective current health, or in the proportion of women who had experienced hot flashes, but interested women more frequently reported depression and sleeplessness.

**Table 3:** Comparison of health habits and health of women interested and non-interested in participating in a randomized PHT preventive trial in Estonia, proportion (%) of women, and age-adjusted odds-ratios (OR).

	Interested	Non-interested	OR (95% CI) ^1)^
	(n = 225)	(n = 553)	
	%	%	
Daily smoker	18	14	0.87 (0.56–1.34)
No alcohol	13	20	1.40 (0.88–2.20)
Plenty of exercise in leisure-time	25	28	1.13 (0.78–1.62)
BMI 25–29.9 ^2)^	55	59	0.96 (0.69–1.34)
BMI ≥ 30 ^2)^	26	21	0.68 (0.47–0.99)
Current health good	24	24	1.27 (0.87–1.86)
Some chronic disease	73	67	0.70 (0.49–1.00)
Tiredness	50	45	0.83 (0.60–1.15)
Irritability	29	23	0.80 (0.56–1.16)
Depression	26	19	0.65 (0.45–0.96)
Headache	36	32	0.96 (0.68–1.34)
Sweating	32	27	0.78 (0.55–1.11)
Hot flashes	28	22	0.74 (0.51–1.07)
Sleeplessness	26	20	0.67 (0.46–0.97)
No symptoms	2	9	4.04 (1.57–10.44)

1) Reference group: "interested"

2) kg/m^2^

Fewer women who were interested in participating in the trial agreed that the menopause is a normal phase, and more gave negative aspects of the menopause than did non-interested women (Table 4). A more favourable opinion of PHT was held by interested than by non-interested respondents.

**Table 4:** Comparison of opinions on aging and attitudes to PHT of women interested and non-interested in participating in a randomized PHT preventive trial in Estonia, proportion (%) of women, and age-adjusted odds-ratios (OR).^1)^

	Interested	Non-interested	OR (95% CI) ^2)^
	(n = 225)	(n = 553)	
	%	%	
Menopause is a normal phase	46	56	0.68 (0.50–0.94)
Women do not loose femininity in menopause	52	51	1.01 (0.73–1.39)
			
PHT prevents osteoporosis	27	9	4.27 (2.75–6.64)
PHT should be given to all women with symptoms	32	12	3.20 (2.16–4.74)
PHT should be given to all postmenopausal women	17	3	5.88 (3.18–10.88)
			
Gave positive aspects of menopause	20	17	1.38 (0.92–2.08)
Gave negative aspects of menopause	39	28	1.82 (1.30–2.57)

1) Missing values excluded from the denominator. The proportion of missing values varied from 17 to 21% in the "interested" group, and from 23 to 26% in the "non-interested" group.

2) Reference group: "non-interested"

The "want more information" group was similar to the group of interested women in regard to the variables studied, but they had had fewer gynaecologist appointments in the last 12 months (40% vs. 53%). The "do not know" group exhibited a clearly greater difference from interested women: fewer of them had ever made use of PHT and calcium drugs; they suffered less from irritability, depression, joint pain, sleeplessness, sweating and hot flashes; they had had fewer appointments with gynaecologists because of menopausal symptoms; and they reported fewer both positive and negative aspects of the menopause.

## Discussion

In the case of many background characteristics, the interested and non-interested women were similar to each other, or the differences between them were small. The major differences between them were in age, health, use of health services, experience and attitude towards the menopause. When compared to non-interested respondents, interested women were younger, and they suffered from poorer health in terms of chronic diseases, more reported symptoms, and more visits to a physician. Interested women had more negative experiences with the menopause and a more positive attitude to PHT, and they had also more often used PHT than had non-interested women.

As in previous preventive trials, the interested women were younger than those who were not interested [1]. But in contrast to Britton et al. [1], who found that interested women had a more healthy lifestyle, we did not find differences in health habits – except with regard to overweight. The use of health services positively correlated with women's willingness to join this trial. It seems that women's contacts to health services may increase their willingness to join a trial; or use of health services may result from their poorer health: interested women had more chronic diseases and more symptoms. Or women expected some benefits from the trial. In an imaginary trial of PHT, the advantages that the women expected from the treatment was more important than how benefits were described [16].

Interested women's negative experiences with the menopause and positive attitude to PHT, as well as a positive attitude towards PHT on the part of gynaecologists [17], may have influenced women's interest in the PHT trial. By contrast, a fifth of the respondents had no opinion concerning PHT. Its use is still infrequent in Estonia, and knowledge of PHT is likely to have been scant.

A limitation for generalizing the study results may result from our particular trial design and target group. However, the trial design of blind and non-blind groups was not presented in the invitation letter. Women were not told at this stage of the recruitment which group they would be assigned to, however, they were told of the chance to receive either hormone, placebo or no treatment. This trial concerned only mid-aged women, and different factors may influence men, or young and old people. The instruction to stop filling in the questionnaire if the women had regular menstruation and no use for hormone therapy resulted in mainly postmenopausal women being included in this report; PHT use in Estonia was low at the time of the questionnaire.

When one is recruiting participants for a randomized controlled trial, more information should be obtained from those who do not enter the trial. Ellenberg [18] argues that information involving the selection process should be obtained at each stage of selection, beginning with the screening of potential participants and proceeding to the final enrolment of those who agree to take part. This process may establish some basis for judging limits when one is generalizing results of an intervention trial. In the present population-based study, the characteristics of persons who did not return the questionnaire remain largely unknown. We know that they were somewhat older and were more often residents of the capital than were those who were interested in joining the trial. Only 52% of people living in the capital have Estonian as their home language, and language problems in this area may explain the lower response rate to our Estonian-language questionnaires.

This preventive randomized controlled trial differed from previous preventive trials in that interested women had more chronic diseases and symptoms. In this respect they were more similar to the participants of treatment trials, in which interested persons tend to be sicker rather than more interested. Possibly some women did not regard our trial as a preventive trial but wished for better care or treatment than they would receive outside the trial – as is the case in treatment trials [3]

## List of abbreviations used

Estonian Postmenopausal Hormone Therapy trial EPHT trial

Postmenopausal hormone therapy PHT

Randomized controlled trial RCT

Women's International Study of long-Duration Oestrogen after Menopause WISDOM

Odds ratio OR

Confidence Interval CI

## Competing interests

The author(s) declare that they have no competing interests.

## Authors' contributions

SLH participated in the design and the conducting of the study, acquired and analysed the data, and drafted the manuscript; MH participated in the trial design and gave critical comments on the manuscript; PV participated in the study design; MR participated in the study design and acquisition of the data; EH conceived the study and participated in its design, participated in the acquisition of the data and gave critical comments on the manuscript. All authors read and approved the final manuscript.

## Pre-publication history

The pre-publication history for this paper can be accessed here:


